# Prelimbic and Infralimbic Prefrontal Cortex Interact during Fast Network Oscillations

**DOI:** 10.1371/journal.pone.0002725

**Published:** 2008-07-16

**Authors:** Karlijn I. van Aerde, Tim S. Heistek, Huibert D. Mansvelder

**Affiliations:** Center for Neurogenomics and Cognitive Research (CNCR), Department of Integrative Neurophysiology, VU University Amsterdam, Amsterdam, the Netherlands; University of Southern California, United States of America

## Abstract

**Background:**

The medial prefrontal cortex has been implicated in a variety of cognitive and executive processes such as decision making and working memory. The medial prefrontal cortex of rodents consists of several areas including the prelimbic and infralimbic cortex that are thought to be involved in different aspects of cognitive performance. Despite the distinct roles in cognitive behavior that have been attributed to prelimbic and infralimbic cortex, little is known about neuronal network functioning of these areas, and whether these networks show any interaction during fast network oscillations.

**Methodology/Principal Findings:**

Here we show that fast network oscillations in rat infralimbic cortex slices occur at higher frequencies and with higher power than oscillations in prelimbic cortex. The difference in oscillation frequency disappeared when prelimbic and infralimbic cortex were disconnected.

**Conclusions/Significance:**

Our data indicate that neuronal networks of prelimbic and infralimbic cortex can sustain fast network oscillations independent of each other, but suggest that neuronal networks of prelimbic and infralimbic cortex are interacting during these oscillations.

## Introduction

The medial prefrontal cortex (mPFC) of rodents consists of several areas including the prelimbic and infralimbic cortex [1]–[3]. These adjacent cortical areas have a different cytoarchitecture [2] and partly differ in their connections with other brain areas [3]. Although many studies have addressed the role of prelimbic and infralimbic cortex without distinguishing between the two areas, other studies show specific involvement of prelimbic or infralimbic cortex in cognitive behavior [4]–[6]. Injection and lesion studies in awake animals suggest that the prelimbic cortex is involved in behavioral flexibility [5], [6], whereas the infralimbic cortex seems to be involved in impulsive behavior and habit formation [7]–[10].

High-frequency oscillations in the beta (14–30 Hz) and gamma range (30–80 Hz) have been linked to cognitive processing and working memory in humans [11], [12] and animals [13], [14]. Studies in awake animals show that during working memory acetylcholine levels increase in prelimbic and infralimbic cortex, and that these increased cholinergic levels are necessary for accurate performance [15]–[18]. *In vitro*, cholinergic agonists such as carbachol, induce fast network oscillations in acute slices of rodent cortex [19]–[23]. Despite the distinct roles in cognitive behavior that have been attributed to prelimbic and infralimbic cortex, little is known about neuronal network functioning of these areas, and whether these networks show any interaction during fast network oscillations.

To address these questions, we induced network oscillations using the muscarinic agonist carbachol in acute brain slices of rat prelimbic and infralimbic cortex, while they were connected with each other, or in isolation. We find that fast network oscillations in infralimbic cortex occur at higher frequencies and with higher power than oscillations in prelimbic cortex. The difference in oscillation frequency disappeared when prelimbic and infralimbic cortex were disconnected. Thus, although neuronal networks of prelimbic and infralimbic cortex can sustain fast network oscillations independent of each other, our data suggest that neuronal networks of prelimbic and infralimbic cortex are interacting during these oscillations.

## Results

**Figure 1:**
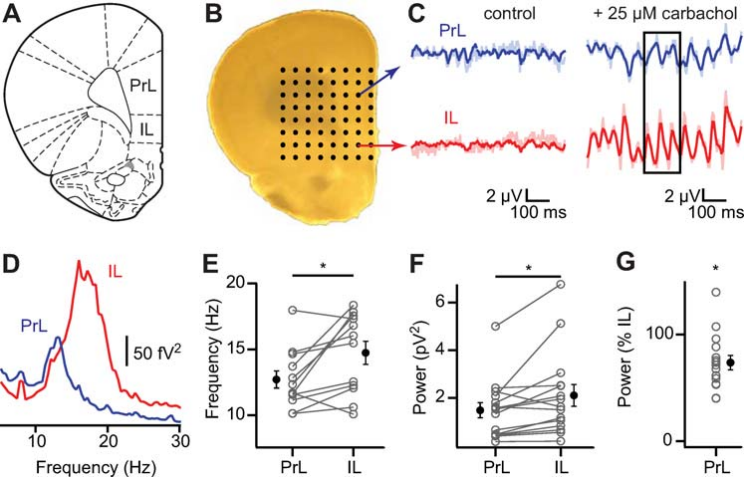
Fast network oscillations are bigger and faster in infralimbic cortex compared to prelimbic cortex. (A) Picture of coronal slice containing medial prefrontal areas prelimbic cortex (PrL) and infralimbic cortex (IL). From The Rat Brain in Stereotaxtic Coordinates by G. Paxinos and C. Watson, 2005. (B) Slice placed on 8×8 multielectrode array. (C) Simultaneous recorded field potentials in prelimbic (top) and infralimbic (bottom) cortex before (left) and after (right) induction of fast network oscillations by carbachol. Bandpass-filtered traces in darker colors. (D) Power spectrum of recordings in (C). (E) Frequency of oscillations in prelimbic and infralimbic cortex (n = 12, *p* = 0.02). (F) Oscillation power (taken as area between 5–35 Hz from power spectrum) (n = 15, *p* = 0.02). (G) Oscillation power in prelimbic cortex as percentage of power in infralimbic cortex (n = 15, *p*<0.01).

To record from prelimbic and infralimbic cortex simultaneously, we placed acute rat prefrontal cortex slices on a planar 8×8 multielectrode grid with an interelectrode distance of 300 µm, covering an area of 2.1 mm^2^ [22], [25] (Figure 1A–C). Bath application of 25 µM carbachol (CCh) induced fast network oscillations that were dependent on glutamatergic and GABAergic transmission (Figure S1). Fourier analysis revealed that field oscillations in the infralimbic cortex oscillated at a higher frequency compared to the prelimbic cortex (Figure 1C–E; mean±s.e.m.; prelimbic (PrL) 12.7±0.7 Hz; infralimbic (IL) 14.7±0.9 Hz; *p* = 0.02, n = 12). Oscillation power in infralimbic cortex was greater than in prelimbic cortex in 13 out of 15 slices, and varied greatly between experiments (Figure 1F; area power spectrum 5–35 Hz; PrL 1.5±0.3 µV^2^; IL 2.1±0.5 µV^2^; *p* = 0.02, n = 15). To calculate the relative power of prelimbic to infralimbic oscillations, we normalized the power in prelimbic cortex to the power in infralimbic cortex per experiment. On average the power in prelimbic cortex was reduced with ∼25% (Figure 1G; PrL 73.7±6.8% of IL, *p*<0.01, n = 15). Thus, fast network oscillations in prelimbic and infralimbic cortex differ in frequency and power.

**Figure 2:**
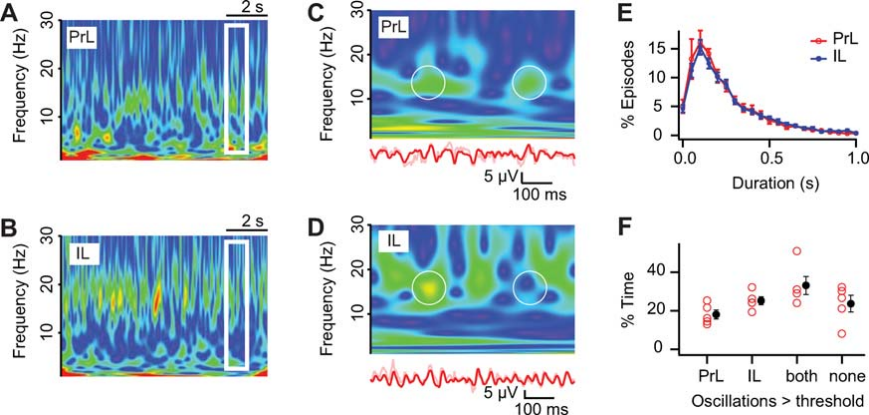
Oscillations in prelimbic and infralimbic cortex can be present simultaneously or separate from each other. (A,B) Frequency fluctuations in time as analyzed with wavelet analysis from simultaneous recordings in prelimbic (A) and infralimbic (B) cortex. Warmer colors represent increasing oscillation magnitudes. (C,D) Expanded time-scale from boxed region in (A,B) with bandpass-filtered (red) field recording (bottom). Circles outline episodes of simultaneous (left circle) and separate (right circle) oscillations. (E) Duration of oscillation episodes in prelimbic (red) and infralimbic (blue) cortex (PrL, n = 5; IL, n = 7; *p* = 0.46). (F) Relative amount of time when oscillations were present in prelimbic and infralimbic cortex simultaneous or separate from each other, or both absent (n = 5).

The power of fast network oscillations strongly fluctuated in time, both in prelimbic and infralimbic cortex. Since fast network oscillations reflect synchronized activity of large groups of neurons [26]–[28], these power fluctuations most likely reflect episodes of increased and reduced synchronicity in neuronal activity, which is a property of neuronal networks [29]. To determine whether neuronal networks in prelimbic and infralimbic cortex show differences in power fluctuations, these fluctuations were quantified using time resolved wavelet analysis [24] (Figure 2A–D). In both prelimbic and infralimbic cortex, the episodes during which the power of oscillations was significantly above threshold (see Materials and Methods, Figure S2) occurred around 2 Hz (PrL 1.9±0.2 Hz, n = 5; IL 1.7±0.1 Hz, n = 7; *p* = 0.59) and lasted about 225 ms (Figure 2E; mean of medians±s.e.m.; PrL 212.9±25.6 ms, n = 5; IL 237.6±20.4 ms, n = 7; *p* = 0.46). The oscillation episodes occurred both simultaneously and separately in prelimbic and infralimbic cortex (Figure 2F; only PrL 18.1±2.3%; only IL 25.2±2.1%; both 33.1±4.7%; neither 23.7±4.3%, n = 5). These findings suggest that fast network oscillations in prelimbic and infralimbic cortex can occur independently from each other.

**Figure 3:**
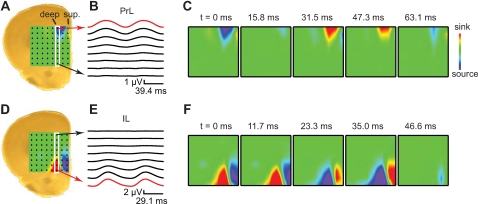
Current source density analysis of prelimbic and infralimbic electrodes reveals two sink-source pairs. (A) Brain slice with superposed 2D-CSD plot as calculated from peak-to-peak cycle averaged field potentials from 8×8 multielectrode array. (B) Peak-to-peak cycle averaged field potentials, using a prelimbic field recording as reference oscillation (red trace). Two oscillation cycles are shown for clarity. The white rectangle in (A) marks the column of 8 electrodes, spanning prelimbic and infralimbic cortex, that are displayed. (C) 2D-CSD plots of 8×8 electrodes at different time points. The CSD plots display alternating sink (red) and source (blue) pairs between deep and superficial layers of prelimbic cortex. Note that sink-source pairs are restricted to prelimbic cortex. (D–F) as (A–C) using an infralimbic field recording (red trace in E) as reference oscillation. Note that sink-source pairs are restricted to infralimbic cortex.

To further investigate whether separate neuronal networks generate fast network oscillations in prelimbic and infralimbic cortex, we analyzed the underlying current sinks and sources that generated the field oscillations with two dimensional current-source-density (CSD) analysis [30] (Figure 3). When an electrode in layer 5 from the prelimbic cortex served as reference electrode, a sink-source pair between layer 5 and the superficial layers 1/2 was revealed (Figure 3C, Movie S1). In 6 out of 12 slices the current sink-source pair was restricted to the prelimbic cortex and did not involve the infralimbic cortex (Figure 3B). When in the same experiment an electrode in layer 5 from the infralimbic cortex served as reference electrode, a current sink-source pair between the deep and superficial layers of infralimbic cortex was revealed (Figure 3F, Movie S2). This sink-source pair did not extend to the prelimbic cortex and was completely restricted to the infralimbic cortex (Figure 3E). These results show that fast network oscillations are generated by separate neuronal networks in prelimbic and infralimbic cortex, and can be restricted to that area.

**Figure 4:**
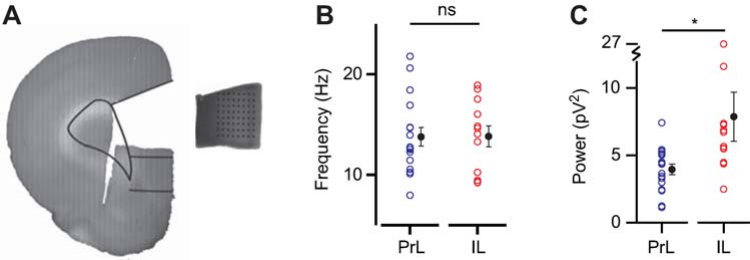
Oscillations in isolated infralimbic mini-slices are bigger but not faster than in prelimbic mini-slices. (A) Mini-slices were cut from coronal slices and placed on a 8×8 multielectrode array. Drawn lines indicate boundaries of prelimbic and infralimbic cortex (adapted from “The Rat Brain in Stereotaxic Coordinates”, by G. Paxinos and C. Watson, 2005). (B) Oscillation frequency of prelimbic and infralimbic isolated mini-slices (PrL, n = 17; IL, n = 12; *p* = 0.97). (C) Oscillation power of prelimbic and infralimbic isolated mini-slices (PrL, n = 17; IL, n = 12; *p* = 0.02).

**Figure 5:**
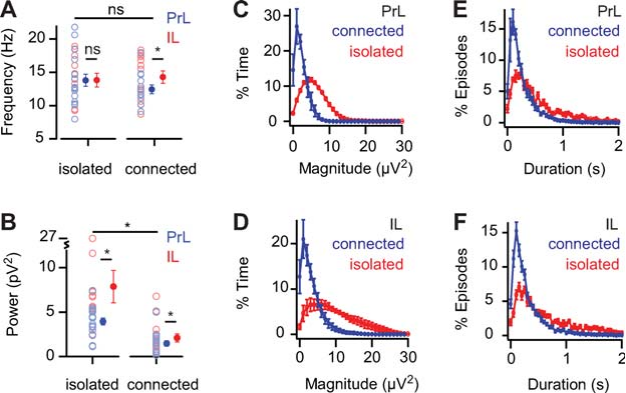
Oscillations are bigger in isolated slices. (A) Frequency of oscillations in isolated and connected slices (PrL: isolated n = 17, connected n = 12, *p* = 0.39; IL: isolated n = 12, connected n = 12, *p* = 0.51). (B) Power of oscillations in isolated and connected slices (PrL: isolated n = 17, connected n = 15, *p*<0.01; IL: isolated n = 12, connected n = 15, *p*<0.01). (C, E) Magnitude distribution and episode duration of oscillations in isolated and connected prelimbic cortex slices (isolated n = 4, connected n = 5, *p*<0.01). (D,F) as (C,E) for infralimbic cortex (isolated n = 5, connected n = 7, *p*<0.01).

Since fast network oscillations in prelimbic and infralimbic are generated by their own neuronal networks, this could suggest that they may exist independent from each other. To test whether neuronal networks from prelimbic and infralimbic cortex can generate and sustain fast network oscillations independent of each other, we cut out mini-slices that included either the prelimbic or the infralimbic cortex (Figure 4A). Application of carbachol to these isolated slice parts induced fast network oscillations in both prelimbic slices and infralimbic slices (Figure 4). The power of oscillations was much larger in both prelimbic and infralimbic isolated slices compared to the slices that contained both areas (Figure 5B; PrL: isolated 4.0±0.4 pV^2^, n = 17, connected 1.5±0.3 pV^2^, n = 15, *p*<0.01; IL: isolated 7.9±1.8 pV^2^, n = 12, connected 2.1±0.5 pV^2^, n = 15, *p*<0.01). This was due to an increase in the median and maximum magnitude of oscillations in isolated slices (Figure 5C, D; median magnitude: isolated-PrL 5.9±0.2 µV^2^, n = 4, connected-PrL 2.5±0.4 µV^2^, n = 5, *p*<0.01; isolated-IL 9.5±1.7 µV^2^, n = 5, connected-IL 3.2±0.6 µV^2^, n = 7, *p*<0.01; maximum magnitude: isolated-PrL 22.1±2.8 µV^2^, n = 4, connected-PrL 10.1±0.8 µV^2^, n = 5, *p*<0.01; isolated-IL 28.8±2.6 µV^2^, n = 5, connected-IL 13.4±2.0 µV^2^, n = 7, *p* = 0.01). Also, the duration of episodes showed a two-fold increase in isolated slices (Figure 5E, F; PrL: isolated 419.6±29.4 ms, n = 4, connected 212.9±25.6 ms, n = 5, *p*<0.01; IL: isolated 576.0±55.6 ms, n = 5, connected 237.6±20.4 ms, n = 7, *p*<0.01). The power of the field oscillations in the isolated infralimbic slices was larger than the power in isolated prelimbic slices (Figure 4C; isolated-PrL 4.0±0.4 pV^2^, n = 17; isolated-IL 7.9±1.8 pV^2^, n = 12; *p* = 0.02), similarly to when the areas were connected (Figure 1F). This suggests that the difference in oscillation power between these areas results from properties within the prelimbic and infralimbic neuronal networks. In contrast, the frequency of oscillations in isolated prelimbic and isolated infralimbic slices was not different (Figure 4B; isolated-PrL 13.8±0.9 Hz, n = 17; isolated-IL 13.8±1.0 Hz, n = 12; *p* = 0.97). This was surprising since the oscillation frequencies were different in prelimbic and infralimbic cortex when these areas were connected (Figure 1D). This could suggest that during fast network oscillations, prelimbic and infralimbic cortical neuronal networks affect each other, giving rise to differences in oscillation frequency, which disappear when these areas are isolated from each other (Figure 5A; PrL: isolated 13.8±0.9 Hz, n = 17; connected 12.7±0.7 Hz, n = 12; *p* = 0.39. IL: isolated 13.8± Hz, n = 12; connected 14.7±0.9 Hz, n = 12; *p* = 0.51).

**Figure 6:**
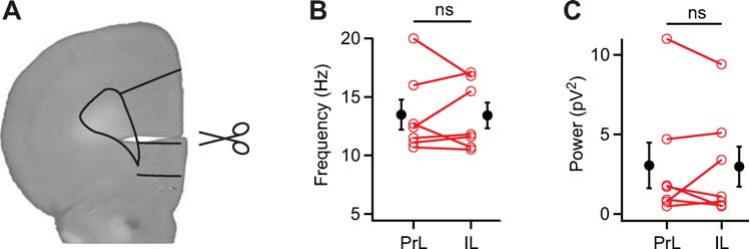
Oscillation frequency difference disappears after disconnecting prelimbic and infralimbic cortex. (A) A cut was made between prelimbic and infralimbic cortex. Drawn lines indicate boundaries of prelimbic and infralimbic cortex (adapted from “The Rat Brain in Stereotaxic Coordinates”, by G. Paxinos and C. Watson, 2005). (B) Oscillation frequency of disconnected prelimbic and infralimbic cortex (n = 7, *p* = 0.94). (C) Oscillation power of disconnected prelimbic and infralimbic cortex (n = 7, *p* = 0.84).

To investigate whether a direct connection between prelimbic and infralimbic cortex modulates the oscillation frequency to be different between these areas, we made a cut in whole coronal slices between prelimbic and infralimbic cortex (Figure 6A). Indeed, after the cut was made there no longer was a difference in oscillation frequency between prelimbic and infralimbic cortex (Figure 6B; PrL 13.5±1.3 Hz; IL 13.5±1.1 Hz; n = 7; *p* = 0.94). Surprisingly, there was also no difference in oscillation power between prelimbic and infralimbic cortex (Figure 6C; PrL 3.1±1.5 pV2; IL 3.0±1.3 pV2; n = 7; *p* = 0.84), which could result from the large variation in oscillation power.

**Figure 7:**
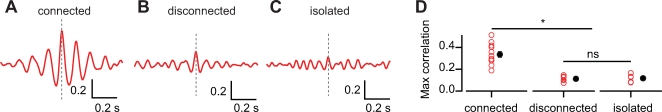
Cross-correlation between prelimbic and infralimbic cortex. (A,B,C) Cross-correlation between prelimbic and infralimbic cortex in a connected slice (A) disconnected slice (B) and between two isolated mini-slices. Vertical dashed line at t = 0 s. (D) Maximal cross-correlation (connected n = 12; disconnected n = 6; isolated slices n = 5, *p*<0.01).

The above results suggest that prelimbic and infralimbic cortex are interacting during oscillations since connected prelimbic and infralimbic cortex show differences in frequency that disappear when the connection between these areas is cut either in whole coronal slices or in isolated mini-slices. Indeed, prelimbic and infralimbic cortex are known to be strongly connected with each other [31]. If these areas are affecting each other during fast network oscillations, one would expect a larger correlation between the field potentials in these areas when they are connected than in isolation. To that end, we cross-correlated the field potentials in connected prelimbic and infralimbic cortex slices, in disconnected whole coronal slices and in slices from isolated prelimbic and isolated infralimbic cortex placed on the same electrode grid (Figure 7). In slices of connected prelimbic and infralimbic cortex there was a significantly higher correlation between the field potentials than when these areas were disconnected or isolated (Figure 7B; cross-correlation at 900 µm; connected: *r* = 0.33±0.03, n = 12; disconnected: *r* = 0.12±0.01, n = 6 isolated: *r* = 0.12±0.02, n = 5; ANOVA *p*<0.01; Newman-Keuls *p*<0.01). This suggests that although infralimbic and prelimbic cortex can generate and sustain fast network oscillations, they do interact and affect synchronization of each others neuronal networks.

## Discussion

Prelimbic and infralimbic cortex are both part of the medial prefrontal cortex [2] and are associated with different aspects of working memory [4]–[10]. Despite the distinct roles in cognitive behavior little is known about neuronal network functioning of these areas, and whether these networks show much interaction during fast network oscillations. We investigated carbachol-induced fast network oscillations in acute rat slices of prelimbic and infralimbic cortex. We performed simultaneous field recordings of connected or disconnected prelimbic and infralimbic cortex, and of isolated prelimbic or infralimbic mini-slices. We found that neuronal networks of prelimbic and infralimbic cortex can sustain fast network oscillations independent of each other in isolated mini-slices. When connected, fast network oscillations in prelimbic and infralimbic cortex remain restricted to their own area in 50% of the slices. Fast network oscillations in the infralimbic cortex displayed a higher power than oscillations in prelimbic cortex, in both connected and isolated slices. In disconnected slices there was no difference in oscillation power, but this could be masked by the large extend of variation.

The difference in oscillation power suggests a difference in internal network properties between prelimbic and infralimbic cortex. The architecture of the microcircuit could play a role in this. In the hippocampus fast network oscillations occur with much higher power, than oscillations generated by neocortical networks [19]–[23]. It is generally assumed that this results from the one layered pyramidal cell structure of the hippocampal circuit [23]. The microcircuit layout of prelimbic and infralimbic cortex is very alike: both consisting of a neocortical multi-layered structure, both lacking layer 4, which is typical of the rodent medial prefrontal cortex [1], [2]. However, there are two striking differences between infralimbic and prelimbic cortical architecture: 1- the lamination in general, and especially of layer 2 and layer 3, is less clear in infralimbic cortex [2], [32]; 2- the prelimbic cortex is thicker and contains a larger number of cells per column than infralimbic cortex [2], [32]. Since fast network oscillations result from the synchronized activity of large groups of neurons [26]–[28], the increased number of cells available in prelimbic cortex would seem an advantage, assuming an equal proportion of cells that participate in fast network oscillations. On the other hand, the thinner cortical layers in the infralimbic cortex could also lead to more alignment of the neurons generating the fast network oscillations, and hence to a greater summation of currents. However, the general decreased lamination of the infralimbic cortex would reduce this effect. If it is not through differences in cell number or lamination, the infralimbic cortex could be more tuned to generate fast network oscillations than prelimbic cortex through other properties, such as possible differences in cell types, intralaminar connectivity or sensitivity to carbachol.

The frequency of fast network oscillations seems to be partly dependent on the interaction between prelimbic and infralimbic cortices. When connected, infralimbic cortex oscillated at a higher frequency than prelimbic cortex. This frequency difference disappeared when prelimbic and infralimbic cortices were disconnected, both in whole coronal slices or in the isolated mini-slices experiments. Cross-correlation analysis of the field potential in prelimbic cortex with the field potential in infralimbic cortex confirmed the interaction between the two areas. Also the power of fast network oscillations was reduced in connected slices compared to isolated slices for both areas, which suggest that the two oscillations could inhibit each other. Prelimbic and infralimbic cortex are intrinsically connected [3], [31]. Prelimbic layer 5/6 projects to infralimbic layer 5/6, and infralimbic layers 1–6 project to primarily to prelimbic layers 1, 3 and 5 [31]. Thus it seems likely that pyramidal cells that fire phase-locked to the local fast network oscillations in one area influence pyramidal cell firing in the other area. How the interaction at the macrocircuit level influences the local fast network oscillations remains an intriguing question. We conclude that neuronal networks of prelimbic and infralimbic cortex can sustain fast network oscillations independent of each other, but do interact during these oscillations and affect synchronization of each others neuronal networks.

The present results suggest that the increase in acetylcholine levels seen in the awake animal during working memory tests and cue detection [15]–[18], have a profound and parallel effect on the distinct network activity in prelimbic and infralimbic cortex. Although, until now, most emphasis has been placed on the role of the prelimbic cortex in behavioral flexibility, the greater response of the infralimbic network to carbachol application would justify more attention for this area. In addition, the connectivity [3], [31] and interaction between prelimbic and infralimbic cortex during fast network oscillations presented here, suggests that these areas could function in concert with each other during high acetylcholine levels.

## Materials and Methods

### Slice Preparation

Prefrontal coronal slices (400 µm) were prepared from P14–28 Wistar rats, in accordance with Dutch license procedures. Brain slices were prepared in ice-cold artificial cerebrospinal fluid (ACSF), which contained: 125 mM NaCl, 3 mM KCl, 1.25 mM NaH_2_PO_4_, 3 mM MgSO_4_, 1 mM CaCl_2_, 26 mM NaHCO_3_, and 10 mM glucose (300 mOsm). Slices were then transferred to holding chambers in which they were stored in ACSF containing: 125 mM NaCl, 3 mM KCl, 1.25 mM NaH_2_PO_4_, 2 mM MgSO_4_, 2 mM CaCl_2_, 26 mM NaHCO_3_, and 10 mM glucose, bubbled with carbogen gas (95% O_2_/5% CO_2_). Slices were left to recover at room temperature for one hour.

### Electrophysiology

After recovery, slices were mounted on 8×8 arrays of planar microelectrodes (electrode size: 50 µm×50 µm; interpolar distance: 150 µm or 300 µm; Panasonic MED-P5155 or MED-P5305; Tensor Biosciences, Irvine, CA). To improve slice adhesion, the multielectrode probes were coated with 0.1% polyethylenimine (Sigma-Aldrich, St. Louis, MO) in 10 mM borate buffer (pH 8.4) for at least 6 hr before use. The multielectrode probe was then placed in a chamber saturated with humidified carbogen gas for at least 1 hr. For recordings, slices were maintained in submerged conditions at 25°C, and superfused with ACSF, bubbled with carbogen, at 4–5 ml/min. Spontaneous field potentials from all 64 recording electrodes were acquired simultaneously at 20 kHz, using the Panasonic MED64 system (Tensor Biosciences), and down sampled off-line to 200 Hz or 2 kHz.

### Data Analysis

Electrophysiological data was analyzed using custom-written procedures in Igor Pro (Wavemetrics, OR, USA).

### Significance of oscillations

After application of 25 µM muscarinic acetylcholine receptor agonist carbachol, neuronal activity in the slice gradually increases (Figure 1C, Figure S2A, B). Power spectrum analysis shows first an increase in 1/f noise during wash-in. Then, after ∼200 sec oscillations occur that show a distinct peak in the power spectrum (Figure S2A, B). Wavelet analysis [24] showed that when the oscillations are clearly present, still the magnitude of oscillations fluctuates in time (Figure 2A–D). To identify and quantify episodes during which field oscillations are present, we compared the wavelet magnitude of ongoing field oscillations with the wavelet magnitude during wash-in period, when the 1/f noise was elevated, but no distinct oscillations were yet visible, i.e. just before the onset of oscillations (time point 128 sec in Figure S2A, B). This prevented false positive identification of oscillation episodes due to the overall increases in 1/f noise.

The onset of oscillations was determined by averaging the wavelet magnitude of 8 sec time windows (but see below) for the whole frequency spectrum: a “global wavelet” (Figure S2A). When followed in time, the global wavelet power spectrum first showed an increase in 1/f noise followed by the appearance of a distinct oscillation peak (Figure S2A). In each experiment, the increase in magnitude at the oscillation frequency was compared to the reference frequency of 5 Hz, the magnitude of which only increased as part of the 1/f noise. The time point of the onset of oscillations was determined as the intersection of these curves (Figure S2B). The 95% confidence interval of an exponential fit to the global wavelet power spectrum at the time window preceding the onset of oscillations was taken as the threshold for oscillations during the entire recording (Figure S2C).

To determine the impact of the time window size on the threshold, we investigated the effect of window size on the two parameters that determine the threshold. Firstly, the threshold will depend on the time point used for constructing the global wavelet power spectrum. Secondly, the threshold will depend on how well the power spectrum before the onset of oscillations was fitted by a mono-exponential function. When the onset time of oscillations was calculated for different lengths of time windows (2–4–8–16–32 s), there was a general trend towards an earlier onset for larger window sizes (Figure S2D). However, the variation in onset time between experiments and between different electrodes from the same experiment by far exceeded the variation due to different window sizes (avg stdev of time windows within experiments: 6.7±0.3 s; avg stdev between experiments 37.3±0.6 s; *p*<0.05). Thus, time window sizes between 2 s and 32 s do not affect the onset time point of oscillations.

In contrast, the goodness of fit of the global wavelet power spectrum was affected by the time window size (Figure S2E). The noise in the global wavelet power spectra obtained with short time window sizes below 8 seconds gives rise to a broad 95% confidence interval of the exponential fit. Plotting the 95% confidence interval of the fit against different time window sizes for different experiments and different electrodes showed that the confidence interval becomes narrower with increasing time window lengths (Figure S2F). Time window sizes above 8 seconds did not result in narrower confidence intervals. To determine the onset of oscillations with sufficient time resolution, but at the same time with sufficiently low noise levels to obtain a good exponential fit, a time window of 8 seconds was used in all experiments.

### CSD

All signals were peak-to-peak averaged relative to a reference recording from layer 5. Signals were band-pass filtered between 5 and 35 Hz before detection of negative signal peaks. Each peak-to-peak signal was interpolated to a 100-point wave, and these waves were averaged to provide the peak-to-peak average. Current source density (CSD) analysis was performed on the peak-to-peak average cycles. For two-dimensional CSD signals were passed through a 3×3 Gaussian spatial filter and convolved with a 3×3 Laplacian kernel (0 −1 0, −1 4 −1, 0 −1 0), as previously described [22], [25]. CSD plots are shown using an inverted colour scale, with warm colours corresponding to current sinks (i.e., neuronal membrane inward currents) and cool colours corresponding to current sources.

### Statistics

Data are represented as mean±SEM. Statistical analysis used either the Student's t test (paired or unpaired) or an ANOVA with Student Newman Keuls *post-hoc* test, as appropriate. Asterisks represent *p*<0.05.

### Drugs and Chemicals

Carbamoylcholine chloride (carbachol, CCh), bicuculline-methiodide and atropine were from Sigma-Aldrich (St. Louis, MO); 6,7-dinitroquinoxaline-2,3(1H,4H)-dione (DNQX) from RBI (Natrick, MA, USA).

## Supporting Information

Figure S1 Pharmacology of fast network oscillations.
(0.70 MB TIF)

Figure S2 Method for calculation of significance of fast network oscillations.
(0.58 MB TIF)

Movie S1 Current-source-density analysis reveals a sink-source pair that is restricted to the prelimbic cortex. CSD movie as calculated from peak-to-peak cycle averaged field potentials, using a prelimbic field recording as reference oscillation, from 8×8 multielectrode array. Two oscillation cycles are shown for clarity. The CSD movie displays an alternating sink (red) - source (blue) pair between deep and superficial layers of the prelimbic cortex. Note that the sink-source pair is restricted to the prelimbic cortex.
(3.67 MB MOV)

Movie S2 Current-source-density analysis reveals a sink-source pair that is restricted to the infralimbic cortex. CSD movie as calculated from peak-to-peak cycle averaged field potentials, using an infralimbic field recording as reference oscillation, from 8×8 multielectrode array. Two oscillation cycles are shown for clarity. The CSD movie displays an alternating sink (red) - source (blue) pair between deep and superficial layers of infralimbic cortex. Note that the sink-source pair is restricted to the infralimbic cortex.
(3.99 MB MOV)
