# Supplementary figures and images for: Prelimbic and Infralimbic Prefrontal Cortex Interact during Fast Network Oscillations
Source: PLoS One. 2008 Jul 16;3(7):e2725. doi: 10.1371/journal.pone.0002725 (PMC2444037; doi:10.1371/journal.pone.0002725)

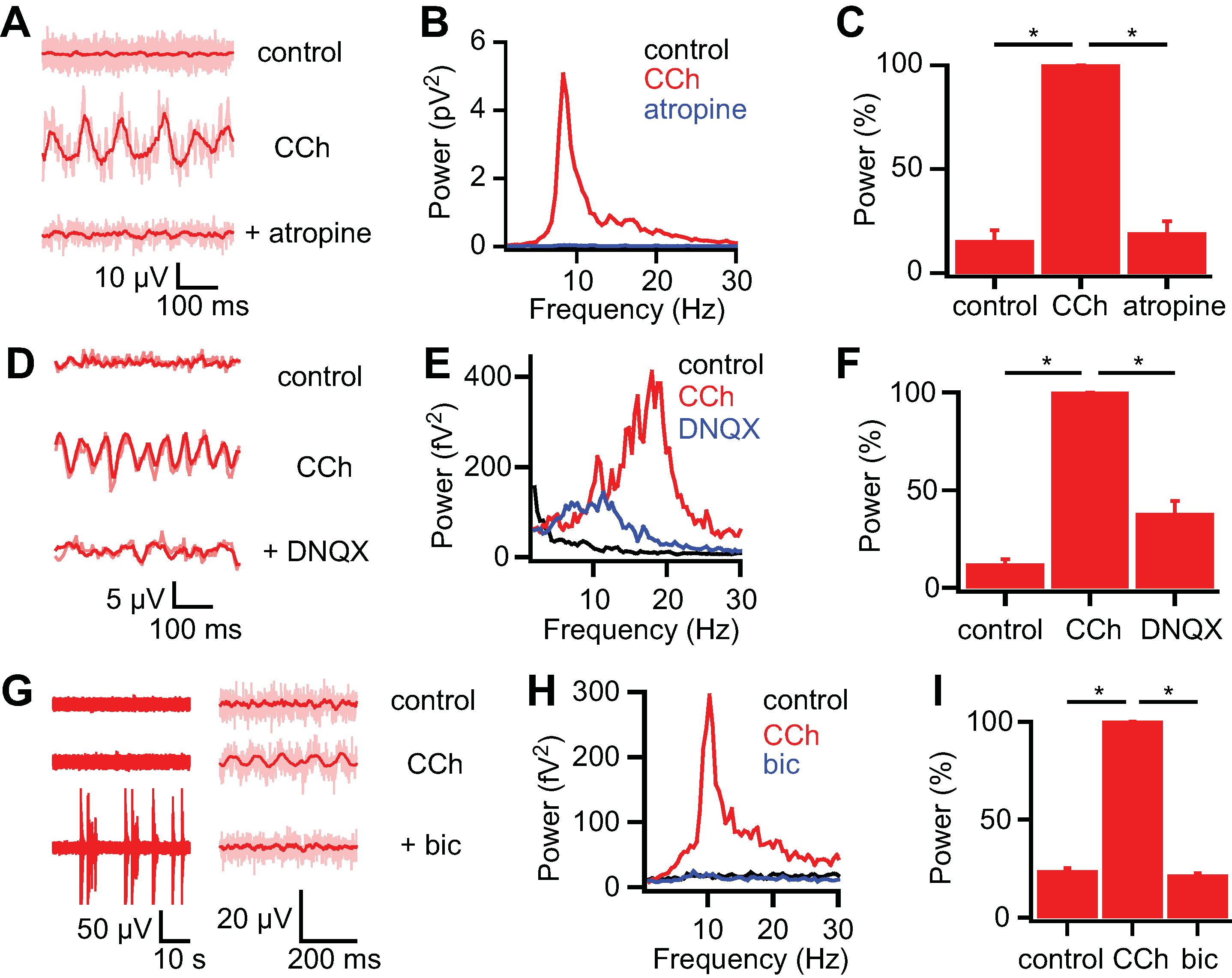

Supplement: Figure S1 — Pharmacology of fast network oscillations. (0.70 MB TIF) [file pone.0002725.s001.tif]

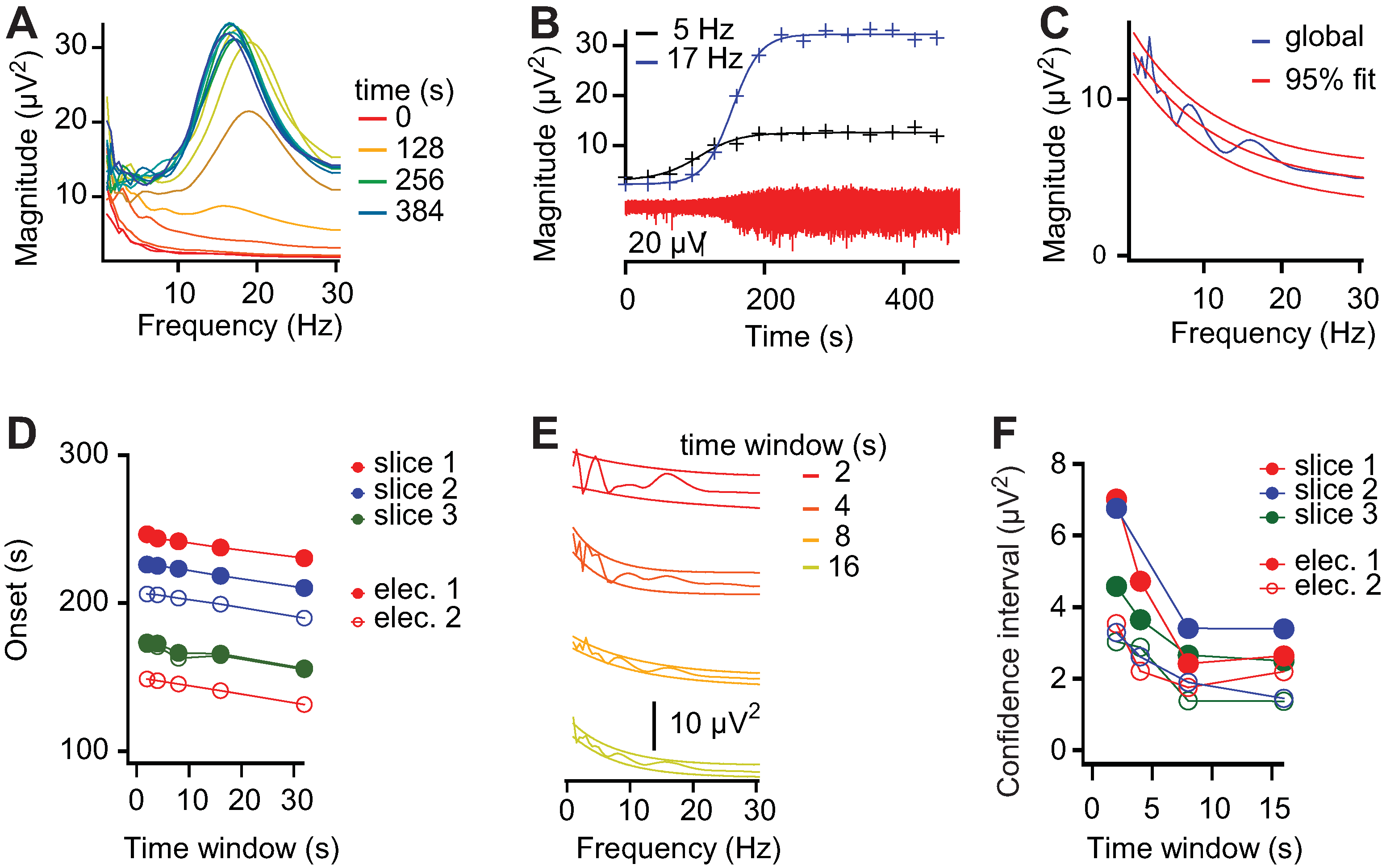

Supplement: Figure S2 — Method for calculation of significance of fast network oscillations. (0.58 MB TIF) [file pone.0002725.s002.tif]
